# Evaluation of Loss Due to Storm Surge Disasters in China Based on Econometric Model Groups

**DOI:** 10.3390/ijerph15040604

**Published:** 2018-03-27

**Authors:** Xue Jin, Xiaoxia Shi, Jintian Gao, Tongbin Xu, Kedong Yin

**Affiliations:** 1 School of Economics, Ocean University of China, Qingdao 266100, China; jinxue@ouc.edu.cn (X.J.); shixiaoxia@stu.ouc.edu.cn (X.S.); gaojintian@ouc.edu.cn (J.G.); xutongbin@stu.ouc.edu.cn (T.X.); 2 College of Oceanic and Atmospheric Sciences, Ocean University of China, Qingdao 266100, China; 3 Ocean Development Research Institute, Major Research Base of Humanities and Social Sciences of Ministry of Education, Ocean University of China, Qingdao 266100, China

**Keywords:** storm surge disaster, loss evaluation, grade classification, econometric model groups, support vector machines

## Abstract

Storm surge has become an important factor restricting the economic and social development of China’s coastal regions. In order to improve the scientific judgment of future storm surge damage, a method of model groups is proposed to refine the evaluation of the loss due to storm surges. Due to the relative dispersion and poor regularity of the natural property data (login center air pressure, maximum wind speed, maximum storm water, super warning water level, etc.), storm surge disaster is divided based on eight kinds of storm surge disaster grade division methods combined with storm surge water, hypervigilance tide level, and disaster loss. The storm surge disaster loss measurement model groups consist of eight equations, and six major modules are constructed: storm surge disaster in agricultural loss, fishery loss, human resource loss, engineering facility loss, living facility loss, and direct economic loss. Finally, the support vector machine (SVM) model is used to evaluate the loss and the intra-sample prediction. It is indicated that the equations of the model groups can reflect in detail the relationship between the damage of storm surges and other related variables. Based on a comparison of the original value and the predicted value error, the model groups pass the test, providing scientific support and a decision basis for the early layout of disaster prevention and mitigation.

## 1. Introduction

Storm surges are abnormal coastal sea-level events caused by meteorological conditions, such as tropical cyclones. In general, the storm surge is an abnormal rise of sea water associated with low pressure weather systems, such as tropical cyclones (typhoons) and strong extra tropical cyclones [1,2]. Storm surges are by far the most damaging marine disaster [3,4]. Most of the world’s great coastal disasters are caused by storm surges [5]. Estimates are based on data from the International Disaster Prevention and Mitigation Commission and the Emergency Events Database. From 1949 to 2016, global marine disasters occurred 2757 times—storm surge disasters occurred 1562 times, which accounts for 56.66% of the total. The direct economic loss caused by storm surges was as high as $4951.85 billion. With the rapid development of China’s economy and the increasing level of urbanization [6], coastal cities are becoming regional economic development centers [7]. Coastal areas bear a large proportion of population and economy and are exposed to typhoons and storm surges [8]. Storm surges have the potential to cause loss of life and financial damage [9,10] and have done so on many occasions in the past [11,12]. Globally, China is one of the countries most frequently and severely affected by storm surge disasters [13,14]. During the period of 1949–2016, storm surges have occurred 602 times in China, resulting in direct economic losses of up to 313.64 billion yuan. They have occurred 160 times in the last 20 years, accounting for more than 90% of all marine disaster loss. Storm surges not only threaten safety but also cause property damage, so it is necessary to use scientific means to evaluate storm surge disaster loss [15]. Assessing the loss due to storm surges can provide scientific support and a decision-making basis for the early layout of disaster prevention and mitigation [16].

Studies using model and quantitative methods for disaster damage assessment have begun, and many results have been found, but there has been little research on the damage assessment of storm surges. In 1992, the Sea, Lake and Overland Surges from Hurricanes (SLOSH) model was used for the first time to estimate storm surge disaster loss in the United States. Using geographic information system (GIS) to input water depth data and ground digital elevation data, the storm surge disaster risk area was determined to estimate the loss due to storm surges [17]. In 1997, a 7-step vulnerability assessment method proposed by the International Panel on Climate Change (IPCC) established an evaluation index system from five aspects: social and economic loss, the loss of ecosystems, and the loss of cultural and historical heritage [18]. In 2003, the US Federal Emergency Management Agency (FEMA) and the National Institute of Building Sciences (NIBS) developed a multi-hazard loss assessment model HAZUS-MH, which focused on three types of disasters: earthquakes, hurricanes, and floods [19]. In 2003, the United Nations Economic Commission for Latin America and the Caribbean (UN-ECLAC) presented a set of Economic Commission for Latin America and the Caribbean (ECLAC) assessment methods to assess the socio-economic impacts of natural disasters. The assessment of natural disaster loss was combined with the long-term social and economic development plan of the country (region) [20].

Narayan [21] used the computable general equilibrium model to assess the damage of tropical cyclone disasters and to study the impact of disaster loss on short-term macro economy. Rose et al. [22] built a computable general equilibrium model for disaster impacts, assessing the indirect economic loss of the associated departments and associated areas caused by an earthquake disaster in Portland, USA. Based on the static input–output model, the concept of a time series was employed, and a dynamic input–output model was constructed to evaluate the indirect economic loss caused by natural disasters [23]. A modeling framework based on an input–output table has been presented to study the consequences of natural disasters during the reconstruction phase [24]. By summarizing the work done in the past few decades, Erdik et al. [25] established a new seismic response system function to estimate the loss time of a hazard bearing body after the earthquake. A DPSIR (drivers, pressures, state, impact, response model of intervention) framework [26] has been used for the analysis of the vulnerability of coastal hotspots [27]. Hayashi [28] has argued that a quick assessment of economic loss after any natural disaster is impossible without a post-disaster reconstruction plan and financial budgets. The loss to residential construction from both wind and surges has been estimated via assembly-based vulnerability [29]. Czajkowski et al. [30] has determined tropical cyclone inland flooding loss on a large scale through a new methodology based on flood peak ratio. The transport model proposed by the United Stated Department of Transportation and the Metropolitan Planning Organization has been used to estimate the indirect economic cost of the hurricane disaster [31]. Kim et al. [32] has used a GIS to combine and produce spatial data and a multiple regression method to establish a wind damage prediction model. The expected damage function method is a methodological approach for estimating ex ante reductions in coastal communities [33]. Yang et al. [34] utilized the extended Kalman filter method to project essential property loss and loss of lives based on field observations and measurement indicators. The proposed grey relational model based on dispersion of panel data has been used to research storm-tide disaster loss in China’s coastal [35]. A statistical model has been designed using extreme value distribution theory to measure the adjusted storm surge disasters’ direct economic loss and the return periods for the highest surge levels [36].

Current disaster prevention and mitigation measures against storm surges are generally divided into two aspects: non-engineering measures and engineering measures. Non-engineering measures include formulating emergency rescue programs, the rational utilization of coastal resources, the strengthening management of coastal areas, and disaster monitoring and forecasting systems [4]; engineering measures include strengthening the construction of storm surge coastal monitoring stations, increasing the embankment construction, building mangroves and sand belts, and combining engineering measures with biological measures to form a system to protect against storm surge disasters [13,14,37].

Improving the optimization of a standard formative storm surge evaluation system and building storm surge disaster loss assessment model groups are important parts of storm tide disaster loss assessment. The implementation process should be based on an analysis of the typical regional social and economic structure, the natural environmental structure, disaster loss factors, and a fine classification of the types of disaster loss. At present, the classification of storm surge damage is not unified nor meticulous. The assessment object of disaster loss is relatively vague and the evaluation area is relatively large and rugged. Most of the existing loss assessment models are based on foreign practice. The evaluation model is single and the evaluation conclusion is quite different. A model of joint assessment has not yet been constructed, and the adaptability of the loss assessment model has not been tested. In particular, study on the loss due to storm surges based on natural attributes and socio-economic attributes is lacking.

In order to solve these problems, two new ideas are brought forth to the study of storm surge disaster loss in this paper. Firstly, considering the intensity and situations of disaster formation, based on the eight kinds of storm surge disaster hierarchical approaches, a new method for the classification of typical storm surge disasters is established. Secondly, a method of model groups is proposed for the first time to study storm surge damage. The quantitative relationships between influencing factors and dependent variables are discussed by means of building submodules. The support vector machine (SVM) model is used to verify the rationality of the model groups.

The remainder of the thesis is structured as follows. In Section 2, we comprehensively analyze related research methods and classify disasters after considering the intensity and conditions of the disaster. In Section 3, we design the storm surge disaster loss measurement model and conduct an Augmented Dickey–Fuller test (ADF) test for model group variables and a covariance test for equation variables. An eight-equation storm damage assessment model that reflects the relationship between the breakdown of various storm surge disasters and their natural and economic influencing factors is constructed in Section 4. Section 5 uses the SVM model for loss assessment and sample prediction to test the accuracy of the econometric model groups. Finally, Section 6 summarizes significant findings and describes possibilities and requirements for future research.

## 2. Storm Surge Disaster Intensity Grade Division

Storm surge intensity grading refers to storm factor indicators and usually includes storm surge elevation, the warning water level for storm surge prevention, and submerged depth. According to the intensity of the disaster factor, the storm disaster level can be divided into 4–5 levels [38].

### 2.1. Storm Surge Elevation 

The storm surge elevation is an important indicator of the typhoon disaster level and has been used by the Saffir–Simpson Team [39] in storm surge classification. Guo et al. [40] also shared similar views. The disaster intensity division based on storm surge elevation is shown in Table 1.

### 2.2. The Hypervigilance Tide Level

The inundation of storm surges is finally reflected in the flood prevention and protection capabilities after the water level exceeds the coast. The disaster intensity grade of storm surges based on a super warning tide is common, as shown in Table 2.

### 2.3. Storm Surge Disaster Loss

The magnitude of the intensity of a storm surge can affect the size of the storm surge, and the magnitude of storm surge disaster loss can as well. Storm surge risk and disaster loss are not necessarily proportional. Disaster damage caused by storm surges is not necessarily positively correlated with intensity of hazards, and vice versa. As shown in Table 3 and Table 4, the magnitude of the disaster is included in the disaster loss classification criteria [46,47]. The economic and social impact of storm surge disasters can be fully taken into account based on the classification of storm surge disaster loss.

### 2.4. Classification of Storm Surge Disasters

The province of Guangdong is one of the areas most seriously affected by storm surges in China. As per the statistics of China Marine Disaster Bulletin (2000–2016), we classify storm surges into eight categories based on their intensity. The results are shown in Table 5.

## 3. Storm Surge Disaster Loss Measurement Model Groups 

### 3.1. Model Groups Structure Module Design

According to the characteristics of storm surge disaster loss, the variables of the storm surge disaster loss measurement model cover six major parameters: agricultural loss, fishery loss, human resource loss, the loss of engineering facilities, the loss of living facilities, and direct economic loss. According to the mature experience at home and abroad, the six structural modules of the storm surge hazard measurement model groups were designed and constructed with reference to the structure of the macroeconomic econometric model groups.

(1) The agricultural loss module mainly considers the beach area and thus considers farmland submerged and crop damage.
(2) The disaster fishery loss module considers damage caused by the tide of the storm surge, which affects the beach and the beach area, and thus damage to the aquaculture area resulting in disruption with respect to fishes, shrimps, shellfishes, kelps, and other aquaculture. In addition, waves will cause ships to collide, become stranded, become damaged, or sink, thus causing fishery damage.
(3) The human resource loss module considers the threat to human lives and property and thus considers the affected population, resulting human deaths, missing persons, and the resettlement population.
(4) The engineering facility loss module considers damage to piers, seawalls, revetments, roads, and bridges.
(5) The living facility loss module, which includes extreme weather caused by the storm surge, considers damage to housing, vehicles, and living facilities (household appliances and furniture).
(6) The economic loss module considers data derived from the calendar year China Marine Disaster Bulletin.

### 3.2. Variables Test of Storm Surge Disaster Loss Measurement Model Groups 

#### 3.2.1. Variable Selection Instructions

According to the design of the model groups and the availability of the data, the dependent variables of the model group equations include eight areas: agricultural loss, aquaculture loss, fishing vessel damage, affected population, emergency resettlement population, damaged breakwater, damage to the house, and direct economic loss. The main indicators of the storm surge disaster include the center pressure, the maximum wind speed (m/s), the maximum storm increase (cm), the threshold (cm), the highest tide (cm), the maximum wave height (m), and the maximum daily cumulative rainfall (cm). 

Although the index data of the natural attribute are related to the magnitude of the storm surge disaster, these indicators are discrete and irregular. The differential sequence of first and second orders is not stable at the 10% significance level in the variable ADF test of the storm surge natural attribute index data and the storm surge disaster loss. The covariance test between the variables fails. Therefore, the intensity of the disaster-causing factor and the situation of the disaster are taken into account, and the storm surge intensity index is introduced to describe the characteristics of storm surge hazard.

Combined with data and data analysis, the independent variables of the model group equations include not only the storm surge intensity index but also the related variable index, which is closely related to the loss of each module. For example, the agricultural loss module variables selected storm surge intensity and the percentage of agricultural output value in Gross Domestic Product (GDP). Other modules of the equation-dependent variables will be detailed described, combined with the disaster loss assessment of the measurement model groups’ specific equation construction.

#### 3.2.2. Augmented Dickey-Fuller Test of Model Groups Variables

Economic time series data are often non-stationary, and the main economic variables (such as consumption and income) tend to show a consistent upward or downward trend. Maintaining the smoothness of the time series is a prerequisite for other statistical tests. The ADF method is a general method for verifying the smoothness of time series.

Suppose the random model expression:

(1) $$X_{t}=\rho X_{t-1}+\mu_{t}$$

where *μ_t_* is white noise. The random walk sequence *X_t_* = *X_t_*_−1_ + *μ_t_* is nonstationary, which is the case when the parameter *ρ* = 1 in the random Equation (1). If the regression result *ρ* = 1 is found, the variable *X_t_* will have a unit root, which leads to an unstable random walk sequence.

The difference form of Equation (1) is as follows:

(2) $$\Delta X_{t}=\left(\rho -1\right)X_{t-1}+\mu_{t}=\delta X_{t-1}+\mu_{t}$$

We usually use the *t*-test based on the ordinary least squares (OLS) method to determine whether Equation (2) satisfies *δ* = 0 and then determine whether there is a unit root in Equation (1). The t-statistic follows the Dickey–Fuller distribution.

In the actual inspection process, the time series data and the random perturbation term may not satisfy the basic assumptions, which will lead to the failure of the DF test. Dichey and Fuller improved the DF test and proposed the ADF test. The modelare as follows:

(3) $$\mathrm{Model}\;1:\;\Delta X_{t}=\delta X_{t-1}+\sum_{i=1}^{m}\beta_{i}\Delta X_{t-1}+\varepsilon_{t}$$

(4) $$\mathrm{Model}\;2:\;\Delta X_{t}=\alpha +\delta X_{t-1}+\sum_{i=1}^{m}\beta_{i}\Delta X_{t-1}+\varepsilon_{t}$$

(5) $$\mathrm{Model}\;3:\;\Delta X_{t}=\alpha +\beta t+\delta X_{t-1}+\sum_{i=1}^{m}\beta_{i}\Delta X_{t-1}+\varepsilon_{t}$$

The original hypothesis is H_0_: *δ* = 0; that is, there is a unit root. The actual inspection process is carried out in the order of Model 3 → Model 2 → Model 1. If the result of the test is that there is no stationary sequence of the unit root, the test will be stopped. Otherwise, it will continue to be tested until the end of the test of Model 1. The ADF test is an improvement and more accurate compared with the DF test, and the current time series smoothness test is basically based on the ADF method.

The ADF test results of the loss measurement model’s relevant variables are shown in Table 6. The *p*-value of the ADF test was greater than 0.1 at the 10% significance level, while the first order differential sequence of the model group variable was passed at the 10% significance level. Thus, the covariance test can be used to further study the long-term relationship between variables.

#### 3.2.3. Covariance Test for Equation Variables

ADF test results often find the instability of time series, so two non-stationary variable relationship models cannot be built. If the two times series are not stable, but the single order is of the same order, then the two may have a long-term equilibrium relationship, which is the purpose of the co-integration test. The Engle–Granger (EG) test is suitable for a two-variable cointegration test, while the Johansen test (JJ test) can be used for a multivariate cointegration test.

The EG test is carried out in two steps. First, the OLS method is used to estimate the Equation (6):

(6) $$\hat{Y_{t}}=\hat{\alpha_{0}}+\hat{\alpha_{1}}X_{t},e_{t}=Y_{t}-{\hat{Y}}_{t}$$

Then, the ADF method is applied to test the smoothness of *e_t_*. The JJ test of multivariate cointegration is a multi-cointegration test method based on a vector autoregressive (VAR) model.

The results of the covariance test of the variance of the storm surge are shown in Table 7. *p*-values are less than 0.1, indicating that there is long-term stable relationship between the equations in each module.

## 4. Assessment of Storm Surge Disaster Loss Measurement Model Groups

Storm surge disaster loss measurement model groups are designed to reflect the quantitative relationship between the variables of storm surge disasters. Based on the experience of the mature metering model groups at home and abroad, the storm surge disaster loss measurement model groups, which are divided into six modules and include a total of eight equations, were established. The samples were selected from 2000 to 2016, the storm surge process data in Guangdong. The intensity of the storm surge, the intensity of the disaster risk factor, and the disaster situation are taken into account; i.e., the disaster intensity is classified according to the storm surge, the tide level, and the storm surge disaster. At the same time, this paper assumes that the disaster factor and the disaster situation in the measure of storm surge intensity are of the same degree of contribution. In addition, according to the State Oceanic Administration, Marine Monitoring & Forecasting Center of Zhejiang Province, and the majority of experts and scholars’ division, the most serious storm surge disaster is an I-class disaster, and the loss due to storm surges steadily declines as the hierarchy rises. This article also follows this practice; that is, the intensity of storm surges (SSQD) increases, indicating that the intensity of storm surge damage decreased.

### 4.1. The Agricultural Loss Module

We chose the submerged farmland output value, combined with data availability, as the measure of agricultural loss due to storm surge disasters. Flooded farmland output equals submerged farmland, accounting for the proportion of total farmland area, multiplied by the total agricultural output value of GDP. 

The agricultural loss equation includes three variables: drowned farmland output, storm surge intensity, and total agricultural output value. In order to eliminate the impact of inflation, submerged farmland output and agricultural output are measured by their share of GDP.

The agricultural loss equation is:

NYS = 0.0084 − 0.013 × log(SSQD) + 0.155 × NYCZ − 0.142 × AR(1) − 5.514 × MA(1)(7)

*R*^2^ = 0.9986, DW = 1.9019, F-statistic = 177.8792, Prob(F-statistic) = 0.0562. (8)

Note: As described in Table 8, *R*^2^ is R-squared, DW is Durbin-Watson stat.

NYS is the submerged farmland output value, accounting for the proportion of GDP; SSQD is the storm surge intensity; NYCZ is the total agricultural output value of GDP.

The results of the estimation of the agricultural loss equation are shown in Table 8. The *p*-value is less than 0.1; the result is significant.

According to the results of the OLS methodSSQD and the last period’s submerged farmland output value accounting for the proportion of GDP have a negative effect on the current submerged farmland output value accounting for the proportion of GDP (NYS) at a 5% significance level, while the total NYCZ shows the opposite. This means that, when the storm surge takes place, the higher the current agricultural output value is, the greater the submerged farmland loss will be. An increase in the SSQD indicates a decrease in the storm surge loss intensity and submerged farmland loss. Citizens may be afraid of the loss caused by the last storm surge and take precautions in advance to reduce the probable loss of the next occurrence, which may reduce the agricultural loss in the following storm surge disaster. 

### 4.2. The Fishery Loss Module

The fishery loss module includes the aquaculture loss equation and the fishing vessel damage equation.

The aquaculture loss equation is:

SCS = 62.491 − 3.053 × SSQD + 0.092 × SCMJ − 0.370 × AR(2)(9)

*R*^2^ = 0.8844, DW = 2.0109, F-statistic = 10.1978, Prob(F-statistic) = 0.0241. (10)

SCS is the aquaculture-affected area; SSQD is the storm surge intensity; SCMJ is the aquaculture area.

The results of the estimation of the aquaculture loss equation are shown in Table 9. The *p*-value is less than 0.1; the result is significant.

Based on the results of the OLS method, SSQD and the two-period lagged variable of aquaculture-affected area have a negative effect on the current SCS at the 5% significance level, while the SCMJ shows the opposite. This means that, when the storm surge takes place, the larger the current aquaculture area is, the greater the current aquaculture-affected area will be. An increase in the SSQD indicates a decrease in the storm surge disaster loss intensity and the current aquaculture-affected area. 

The model indicates that, by holding the lagged aquaculture-affected area and SCMJ in constant, each unit of SSQD increased will lead to the reduction of 3.053 units in the current SCS. Under the same condition, each unit of SCMJ increased will lead to the reduction of 0.092 units in the current aquaculture-affected area. If the citizens make previsions to reduce the probable loss, the current aquaculture-affected area will reduce by 0.370 units when the two-period lagged variable of aquaculture-affected area rises by one unit.

The fishing vessel damage equation is

SHYC = 12788.52 − 2479.115 × SSQD + 0.097 × YCZS − 0.986 × MA(3)(11)

*R*^2^ = 0.9857, DW = 1.9672, F-statistic = 45.8371, Prob(F-statistic) = 0.0214 (12)

where SHYC is the number of damaged fishing vessels; YCZS is the marine fishery motorized vessel year-end possession (ship).

The results of the econometric model of the fishing vessel damage equation are shown in Table 10. The *p*-value is less than 0.1; the result is significant.

Based on the results of the OLS method, SSQD and marine fishery motorized vessel year-end possession (YCZS) have a negative effect on the current number of damaged fishing vessels (SHYC) at the 5% significance level. That means that, when the storm surge takes place, the larger YCZS is, the greater number of SHYC will be. An increase in the SSQD indicates a decrease in the storm surge disaster loss intensity and damaged fishing vessels. The model indicates that, by holding the three-period lagged variable of random error term and SSQD in constant, each unit of marine fishery motorized vessel year-end possession (YCZS) increased will lead to a reduction of 0.097 units of SHYC. Each unit of SSQD increased will lead to a reduction of 2479.115 units of current damaged fishing vessels. 

### 4.3. The Human Resource Loss Module

The human resource module model mainly consists of the population equation and the emergency transfer resettlement equation.

The affected population equation is

SZBZ = 0.297 − 0.029 × SSQD + 0.0003 × RKMD − 0.815 × AR(2) − 0.999 × MA(13)

*R*^2^ = 0.9339, DW = 2.0030, F-statistic = 17.6660, Prob(F-statistic) = 0.0037. (14)

SZBZ is the proportion of the affected population in the total population; RKMD is the population density.

The results of the econometric model of the affected population are shown in Table 11, and the *p*-value is less than 0.1. The result is significant and the degree of fitting is an improvement.

Based on the results of the OLS method, SSQD and the two-period lagged variable of proportion of the affected population in the total population have a negative effect on the current SZBZ at the 5% significance level, while population density (RKMD) shows the opposite. This means that, when the storm surge takes place, the larger the population density (RKMD) is, the greater the proportion of the affected population in the total population will be. An increase in the SSQD indicates a decrease in proportion of the affected population in the total population. 

The model indicates that, by holding the lagged proportion of the affected population in the total population and SSQD in constant, each unit of population density (RKMD) increased will lead to a reduction of 0.0003 units in the SZBZ. Each unit of SSQD increased will lead to a reduction of 0.029 units in the current proportion of the affected population in the total population. By keeping the current population density (RKMD) and SSQD in constant, each unit of the two-period lagged variable of proportion of the affected population in the total population increased will lead to a reduction of 0.815 units in current proportion, since citizens might make previsions to reduce the loss.

The emergency relocation of the population equation is

AZZY = 64.117 − 12.605 × SSQD − 0.026 × SZRK + 0.092 × RKMD(15)

*R*^2^ = 0.9843, DW = 2.1891, F-statistic = 5.9616, Prob(F-statistic) = 0.0092. (16)

AZZY is the resettlement population; SZRK is the affected population.

The results show that the *p*-value is less than 0.1; the result is significant. The degree of fitting is an improvement.

According to the results of the OLS method, SSQD and SZRK have a negative effect on resettlement population (AZZY) at the 5% significance level, while RKMD shows the opposite effect on the current proportion of the affected population in the total population. This means that, when the storm surge takes place, a reduction in the affected population will increase the current resettlement population. A larger SSQD indicates a decrease in the storm surge disaster loss intensity and the resettlement population. The larger the RKMD is, the greater the resettlement population will be. 

The model indicates that, by holding the affected population and storm surge intensity figure in constant, each unit of RKMD increased will lead to a reduction of 0.0092 units in the resettlement population. By keeping the affected population and population density in constant, each unit of SSQD increased will lead to a reduction of 12.625 units in the resettlement population. By keeping the current population density and storm surge intensity figure in constant, each unit of affected population (SZRK) increased will lead to a reduction of 0.026 units in the resettlement population.

### 4.4. The Engineering Facility Loss Module

Combined with the availability of data, this module considers breakwater damage and seawall damage to examine the loss due to storm surges.

The breakwater damage equation is

FBDH = 180.212 − 48.725 × SSQD + 0.912 × AR(1) − 0.999 × MA(2)(17)

*R*^2^ = 0.9611, DW = 1.8922, F-statistic = 24.7222, Prob(F-statistic) = 0.0129. (18)

FBDH is the damage breakwater length.

The results of the econometric model are as follows in Table 12.

According to the results of the OLS method, SSQD have a negative effect on current damage breakwater length (FBDH) at the 5% significance level, while the period lagged variable of damage breakwater length shows the opposite effect on the current ones. This means that, when the storm surge takes place, the increase in SSQD indicates a decrease in current damage breakwater length. The last period’s damage breakwater length will affect the next period’s previsions, which will further increase the next period’s damage breakwater length.

The model indicates that, by holding the last period’s damage breakwater length in constant, each unit of SSQD increased will lead to a reduction of 48.725 units in the current damage breakwater length. By keeping the storm surge intensity figure in constant, each unit of the next period’s damage breakwater length increased will lead to 0.912 units in current period. 

### 4.5. The Loss of Living Facilities Module

Taking into account the availability of data availability, the residents’ living facility module is examined by house damaged.

The damage to the housing equation is

SHFW = 5.363 − 1.475 × SSQD − 0.935 × MA(3)(19)

*R*^2^ = 0.8875, DW = 1.2293, F-statistic = 11.8349, Prob(F-statistic) = 0.0377. (20)

SHFW is the number of damaged houses.

The results of the estimation of the damage to the housing equation are shown in Table 13. The *p*-value is less than 0.1, and the result is significant.

According to the results of the OLS method, SSQD have a negative effect on current number of damaged houses at the 5% significance level. This means that, when the storm surge takes place, an increase in SSQD indicates a decrease in current number of damaged houses. The model indicates that, by holding the random error terms in constant, each unit of SSQD increased will lead to a reduction of 1.475 units in the current number of damaged houses.

### 4.6. The Direct Economic Loss Module

The direct economic loss equation is

ZSBZ = 0.000.002 × SSQD + 0.002 × JJMD − 0.952 × AR(1) − 0.956 × MA(2)(21)

*R*^2^ = 0.8760, DW = 1.5545, F-statistic = 10.5952, Prob(F-statistic) = 0.0069. (22)

ZSBZ is the proportion of current direct economy loss in GDP; JJMD is the economic density (regional gross national product/area).

The results of the econometric model of the direct economic loss equation are shown in Table 14, and the *p*-value is less than 0.1. The result is significant and the degree of fitting is good.

According to the results of the OLS method, SSQD and the proportion of the last period’s direct economy loss in current GDP have a negative effect onZSBZat the 5% significance level, while JJMDshows the opposite effect. This means that, when the storm surge takes place, the larger the economy intensity is, the greater the direct economy loss will be. An increase in s SSQD indicates a decrease in the storm surge disaster loss intensity. If the citizens make previsions, the probable loss of the next occurrence will be reduced.

The model indicates that, by holding the lagged direct economy loss and economy intensity in constant, each unit of SSQD increased will lead to a reduction of 0.002 units in current direct economy loss. By keeping the lagged direct economy loss and storm surge intensity figure in constant, each unit of JJMD increased will lead to a reduction of 0.002 units in current direct economy loss. By keeping the economy intensity and storm surge intensity figure in constant, each unit of the last period’s direct economy loss increased will lead to a reduction of 0.952 units in current direct economy loss. 

## 5. Prediction of Storm Hazard Disaster Based on Support Vector Machine

There are two essential elements in storm surge disaster prevention and mitigation, one is the prediction of the effective scale of storm surges before the disaster takes place, and the other lies in the disaster assessment of the actual loss due to storm surges. With the gradual informatization of disasters, people are no longer satisfied knowing only the level of storm surge disasters. Weather forecast data does not easily yield storm surge intensity information. To achieve an objective, accurate, and effective storm surge disaster loss value, forecast has become an urgent need. Domestic and foreign scholars have made a substantial amount of research on the prediction method: regression analysis, exponential smoothing, Bayesian Value at Risk (BVAR) model, and so on have been applied by some scholars to carry out forecast analysis; some scholars choose to use the combination forecasting method. However, these methods can only construct time series or regression models based on the causal link of data, without regard to complex characteristics and the internal structure of the data, which leads to massive data information loss. The research shows that the data fitting ability of modern scientific methods and techniques based on artificial intelligence is strong under the premise of sufficient sample data.

### 5.1. Support Vector Machine Model Construction

Support vector machine is a new type of machine learning method, based on statistical learning theory, constructed by Bell Labs and its research team. Thus, SVM can be divided into two categories according to different applications: support vector regression (SVR) and support vector classification (SVC).

Let the training sample vector be $\{\left(x_{1},y_{1}\right),\left(x_{2},y_{2}\right),\ldots \ldots ,\left(x_{k},y_{k}\right)\}$ $x_{i}\in R^{n}\;y_{i}\in R^{n},$ i=1,2,……k . Using the SVM method, a non-linear mapping high-dimensional feature space must be constructed. In this high-dimensional space, the data *x*_i_ is mapped to F to construct an optimal linear regression function:

(23) f(x)=ωφ(x)+b

where ω and φ(x) are the m-dimensional vector, and *b* is the offset. Different from the traditional principle of risk minimization principle to determine the parameters, the support vector regression machine applies the structural risk minimization principle to ensure the accuracy of the forecast, namely,

(24) $$\min R_{stv}=\frac{1}{2}{\left\|\omega \right\|}^{2}+c\frac{1}{k}\sum_{i=1}^{k}L_{i}\left\lfloor x_{i},y_{i}-f\left(x_{i}\right)\right\rfloor .$$

$L_{i}\left\lfloor x_{i},y_{i}-f\left(x_{i}\right)\right\rfloor =\max\left\{0_{i}\left|y_{i}-f\left(x_{i}\right)\right|-\varepsilon \right\}$, which represents the insensitive loss function; $\|\omega {\|}^{2}$ represents the complexity of the function, $\frac{1}{k}\sum_{i=1}^{k}L_{i}\left\lfloor x_{i},y_{i}-f\left(x_{i}\right)\right\rfloor$, which represents the average loss on the training set. The constant C is also called the penalty function, which represents the relationship between the mean loss of the training set and the complexity of the function. We solve Equation (8) and convert it into the following optimization problem:

(25) $$\min\frac{1}{2}{\left\|\omega \right\|}^{2}-c\frac{1}{k}\sum_{i=1}^{k}\left(\vartheta_{i}+\vartheta_{i}{}^{*}\right)\;s_{.}t_{.}=\{\begin{array}{c} \omega \varphi \left(x\right)+b-y_{i}\leq \varepsilon +\vartheta_{i}\\ y_{i}-\omega \varphi \left(x\right)-b\leq \varepsilon +\vartheta_{i}{}^{*}\\ \vartheta_{i}:\vartheta_{i}{}^{*}\geq 0 \end{array}.$$

$\vartheta_{i}$ and $\vartheta_{i}^{*}$ are the relaxation of variables, and the interpretation of other variables are equivalent to Equations (7) and (8). Because the ω’ dimension is large, we refer to the strong duality theorem, introduce the Lagrangian multiplier $\alpha_{1}\;\mathrm{and}\;\alpha_{1}^{*}$, construct the Lagrangian function, and transform the optimization problem of Equation (9) to obtain the dual problem with the original problem of SVM. Equation (26) is applied to easily solve this problem:

(26) $$\underset{\alpha ,\alpha_{i}}{\max}\sum_{i=1}^{k}\left[\alpha_{i}^{*}\left(y_{i}-\varepsilon \right)-\alpha_{i}\left(y_{i}+\varepsilon \right)\right]-\frac{1}{2}\sum_{i=1}^{k}\sum_{j=1}^{k}\left(\alpha_{i}-\alpha_{i}^{*}\right)\left(\alpha_{j}-\alpha_{j}^{*}\right)K\left(x_{i}:x_{j}\right)\;s_{.}t_{.}=\{\begin{array}{c} \sum_{i=1}^{k}\left(\alpha_{i}^{*}-\alpha_{i}\right)=0\\ 0\leq \alpha_{i}^{*}:\alpha_{i}\leq \frac{c}{k},i=1,2,\ldots \ldots k \end{array}.$$

$K\left(x_{i}:x_{j}\right)=\varphi \left(x_{i}\right)\cdot \varphi \left(y_{i}\right)$ is the kernel function. The most commonly used optimal solution kernel function is the Gaussian function $K\left(x_{i}:x_{j}\right)=\exp\left(-\frac{{\left\|x_{i}-x_{j}\right\|}^{2}}{\sigma^{2}}\right)$. Given the dual solution $\bar{\alpha }={\left(\bar{\alpha_{1}}:\bar{\alpha_{1}{}^{*}},\bar{\alpha_{2}}:\bar{\alpha_{2}{}^{*}},\ldots \ldots \bar{\alpha_{k}}:\bar{\alpha_{k}{}^{*}}\right)}^{T}$, the regression function can be obtained: $f\left(x\right)=\sum_{i=1}^{k}\left(\bar{\alpha }-\bar{\alpha^{*}}\right)K\left(x_{i}:x\right)+\bar{b}$.

### 5.2. Storm Surge Disaster Loss Index Selection

Based on the previous analysis of the impact of storm surge hazard, disaster relief, and disaster environment, this section takes 21 typical storm surge disasters from 1989 to 2016 as the study sample. In the process of disaster loss prediction, 18 samples were randomly selected as the training set, and the remaining three samples were used as the test set. The sample data was reduced by a rough set, and the data were derived from the China Statistical Report on Marine Disasters (1989–2016) and the Guangdong Statistical Yearbook.

We first discretize the data based on rough set theory and then use the equal frequency binning to carry out the discretization process. The Johnson algorithm and the genetic algorithm are used to reduce processing at the same time. The six storm surge damage forecast indicators—direct economic loss, the maximum storm surge, alarm tide, economic density, per capita GDP, and population density—are then obtained.

### 5.3. Assessment and Prediction of Storm Surge Damage

**Figure 1:**
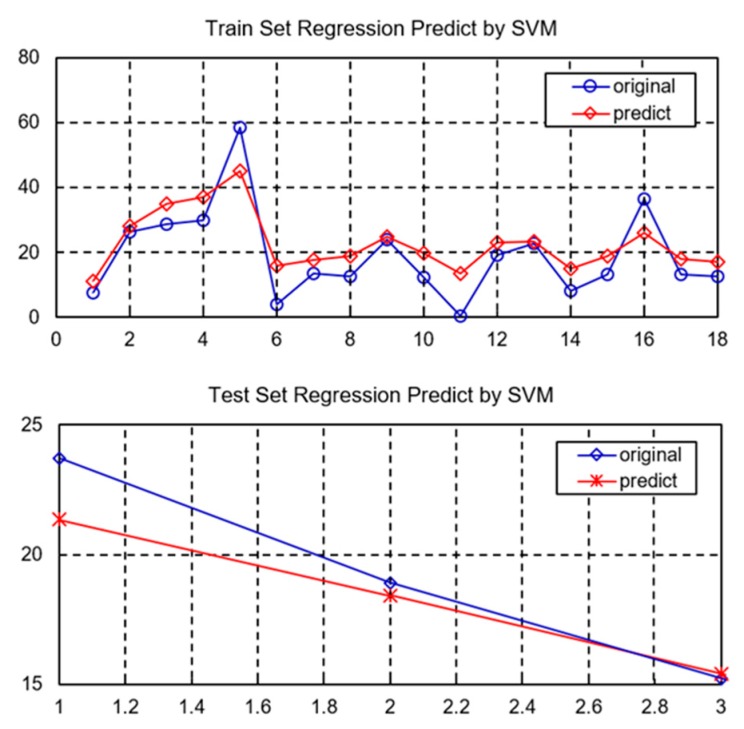
Predictive results of storm surge disasters loss based on support vector machine (SVM).

After determining the optimal parameters of the SVM, this paper makes a fitting prediction of the direct loss of 21 storm surge disasters. Among them, 18 sets of training set data were trained in the study samples, and the remaining three sets of sample data were then predicted. The trend of fitting values and observation values is shown in Figure 1. 

It can be found from the figure that the training effect of the sample and the prediction effect are good, among which the correlation coefficient reaches 0.9921 and the mean square error is only 0.0001. The SVM can obtain a fitting effect in the case of a small sample in the neural network; the fitting degree may be affected when the number of samples is very small. Although the statistical data of storm surges in China is relatively deficient and changes in the statistical caliber are varied, the effect of storm surge disaster prediction is good in the case of small samples. Such a sample is therefore useful as a reference for the prediction of storm surge disasters.

Table 15 shows the prediction of the direct loss of the three storm surge disasters. It can be found that the SVM has an improved prediction effect, and the direct loss of the original value and the prediction error are within the allowable range. Compared with the traditional method based on causality or time series, the SVM can better grasp the intrinsic relationship of the data, grasp the trend of storm surge disasters, and provide scientific support and decision-making basis for disaster prevention and mitigation.

## 6. Conclusions

Most of the world’s great coastal disasters are caused by storm surges. China is one of the countries most frequently and severely affected by storm surge disasters. The index data of the natural properties of storm surges is comprehensively integrated into the index of storm surge disaster intensity in order to prepare for the selection of model group equation variables. By comprehensively considering the situation and intensity of the disaster factors, a typical storm surge disaster can be classified based on eight types of storm surge disaster hierarchical approaches. By combining the characteristics of storm tide disaster loss, we constructed storm surge disaster loss measurement model groups that include eight equations and consist of six major modules: storm surge disaster in agricultural loss, fishery loss, human resource loss, engineering facility loss, living facility loss, and direct economic loss. The model group variable ADF test and equation variable co-integration test were performed, and the results all pass inspection. The model groups reflect the relationship between the damage of different storm surges and the various influencing factors. The statistical data of 21 storms in Guangdong from 1989 to 2016 was selected as research samples. To consider the non-linearity of storm surge data, the SVM model was used to evaluate the loss and the intra-sample prediction. The original value and the predicted value error were controlled within the allowable range.

When a storm surge is coming, related departments can assess the storm surge disaster grade according to the measured data of the storm surge elevation and the hypervigilance tide level. The result of the storm surge disaster grade was brought into the model group structure module equation to roughly estimate the loss of each module. This can provide a scientific basis and decision basis for the early layout of disaster prevention and reduction.

The aim of an assessment of storm surge damage is to achieve a detailed assessment, not only to refine the type of disaster loss but also to subdivide the evaluation object and the assessment area. It is more important to improve and optimize the accuracy of the evaluation model, and to establish model groups for a joint assessment of disaster loss. In the next paper, we will further study the indirect assessment of storm surge disaster loss based on input–output models. There are some deficiencies in the statistics of storm surges in China. If the data were more abundant, the number of equations of storm surge disaster loss measurement model groups could be further expanded. By combining the climatic conditions, natural conditions, sea conditions, sea bearing capacity, and socio-economic structure of typical coastal regions, the regional difference, the type difference, and the time effect of storm surge damage assessment can be reflected. The assessment model groups of disaster loss measurement were constructed based on three levels of module, industry, and region to achieve a detailed assessment of storm surge damage. The aim is to provide authorities with an accurate warning and reduce national disaster.

## Figures and Tables

**Table 1:** Storm surge disaster intensity division based on storm surge elevation (unit: cm).

Grade	State Oceanic Administration (2012) [41]	Gao et al. (2007) [42]	Gan et al. (1991) [43]	Marine Monitoring & Forecasting Center of Zhejiang Province (2011) [44]
1	≥450	≥300	>430	≥251
2	300–450	200–300	231–430	201–250
3	150–300	100–200	131–230	151–200
4	0–150	0–100	≤130	101–150
5		50–100		

**Table 2:** Storm surge disaster intensity division based on the hypervigilance tide level (unit: cm).

Grade	State Oceanic Administration (2011) [45]	Marine Monitoring & Forecasting Center of Zhejiang Province (2011) [42]	Yang et al. (1991) [46]
1	>80	≥151	>200
2	30–80	81–150	>100
3	0–30	31–80	>50
4	−30–0	0–30	Over or near

**Table 3:** Storm surge disaster intensity division based on disaster loss [46].

Grade	Great Tide	Severe Tide	Greater Tide	Mild Tide
Disaster situation	The death of more than a thousand people or economic loss of several hundred million yuan	Hundreds of deaths or economic loss of ¥0.2–1 billion	Dozens of deaths or economic loss of about ¥10 million	No or less death or economic loss less than ¥1 million

**Table 4:** Storm surge disaster intensity division based on disaster loss [47].

Grade	Micro Disaster	Small Disaster	Medium Disaster	Disaster	Catastrophe
Direct economic loss/billion	<10	10–100	100–200	200–400	>400
Affected population/million people	<10	10–100	100–200	200–400	>400

**Table 5:** Classification of storm surge disasters

Numbering	Storm Surge Disaster Intensity Grade Division								
	a	b	c	d	e	f	g	h1	h2
1409	II	I	II	I	II	III	IV	IV	II
1415	I	I	I	I	I	I	II	IV	II
1319	III	II	II	II	II	III	IV	IV	I
1208	III	II	II	II	I	II	II	V	IV
1213	III	II	II	I	I	II	II	IV	III
1117	II	I	II	I	II	III	III	IV	IV
0915	III	II	III	II	I	II	II	IV	III
0814	III	II	II	I	I	I	II	III	I
0601	III	III	III	III	III	IV	IV	IV	I
0516	IV	III	III	IV	II	III	III	V	IV
0307	II	I	II	I	I	II	II	IV	I
0313	III	III	III	III	II	III	III	IV	I

Note: Four divisions are based on the storm surge elevation: a: State Oceanic Administration; b: Gao et al.; c: Gan et al.; d: Marine Monitoring & Forecasting Center of Zhejiang Province. Three are based on the hypervigilance tide level: e: State Oceanic Administration; f: Marine Monitoring & Forecasting Center of Zhejiang Province; g: Yang et al. h1 and h2 are based on storm surge disaster loss and stand for Yang et al. and Zhao et al., respectively.

**Table 6:** Unit root test results of the loss measurement model variables.

Variables	Original Value			First Order Difference		
	t-Statistic	Prob. *	Result	t-Statistic	Prob. *	Result
SSQD	−0.9995	0.2439	unstable	−4.9022	0.0012	stable
NYS	0.9837	0.8821	unstable	−2.5379	0.0229	stable
NYCZ	1.5577	0.9492	unstable	−1.4867	0.0900	stable
SCS	−0.7399	0.3643	unstable	−3.8380	0.0020	stable
SCMJ	−0.9647	0.2735	unstable	−2.7103	0.0146	stable
SHYC	−1.2703	0.1647	unstable	−2.5877	0.0240	stable
YCZS	−0.8137	0.3146	unstable	−2.0348	0.0538	stable
SZBZ	−1.0960	0.2302	unstable	−3.8932	0.0015	stable
RKMD	−1.1299	0.2187	unstable	−2.3295	0.0257	stable
FBDH	−0.8955	0.2938	unstable	−2.8010	0.0137	stable
SHFW	−2.2652	0.2086	unstable	−3.4470	0.0760	stable
ZSBZ	−3.0809	0.1632	unstable	−3.7998	0.0018	stable
JJMD	0.0085	0.9396	unstable	−4.0100	0.0152	stable

Note: SSQD: storm surge intensity; NYS: submerged farmland output value accounting for the proportion of GDP; NYCZ: total agricultural output value of GDP; SCS: aquaculture-affected area; SCMJ: aquaculture area; SHYC: number of damaged fishing vessels; YCZS: marine fishery motorized vessel year-end possession (ship); SZBZ: proportion of the affected population in the total population; RKMD: population density; FBDH: damage breakwater length; SHFW is the number of damaged houses; ZSBZ: proportion of current direct economy loss in GDP; JJMD: economic density. *: 10% significance level.

**Table 7:** The Engle–Granger (EG) cointegration test for the equations of storm surge disaster loss.

Equation	Variable	t-Statistic	Prob. *	Result
Agricultural loss module	NYS and SSQD	−3.1576	0.0745	exist
	NYS and NYCZ	−3.3468	0.0604	exist
Fishery loss module	SCS and SSQD	−2.1581	0.0365	exist
	SCS and SCMJ	−1.1987	0.0938	exist
	SHYC and SSQD	−3.2001	0.0087	exist
	SHYC and YCZS	−2.2721	0.0352	exist
Human resource loss module	SZBZ and SSQD	−2.1008	0.0473	exist
	SZBZ and RKMD	−4.1037	0.0133	exist
Engineering facility loss module	FBDH and SSQD	−3.3422	0.0466	exist
Living facility loss module	SHFW and SSQD	−1.8346	0.0745	exist
Direct economic loss module	ZSBZ and SSQD	−2.8299	0.0855	exist
	ZSBZ and JJMD	−3.3470	0.0409	exist

Note: *: 10% significance level.

**Table 8:** Estimation of the agricultural loss equation measurement model.

Dependent Variable: NYS				
Variable	Coefficient	Std. Error	t-Statistic	Prob.
C	0.008433	0.011412	0.738912	0.0595
LSSQD	−0.012609	0.007795	−1.617580	0.0353
NYCZ	0.154882	0.107694	1.438178	0.0387
AR(1)	−0.141919	0.546633	−0.259624	0.0383
MA(1)	−5.513747	6.082988	−0.906421	0.0312
R-squared	0.998597	Mean dependent var		0.002924
F-statistic	177.8792	S.D. dependent var		0.002671
Prob(F-statistic)	0.056168	Durbin-Watson stat		1.901927

Note: R-squared describes the fitting degree between the model and the sample, and it is better if the R-squared is closer to 1; Mean dependent var describes the mean value of the dependent variables; S.D. dependent var describes the standard deviation of dependent variables; F-statistic describes the overall significance level of the model; Durbin-Watson stat (statistic) is used to test whether the distribution of residuals is a normal distribution, and the model has a strong explanatory power if the DW is about 2.

**Table 9:** Estimation of the aquaculture loss equation measurement model.

Dependent Variable: SCS				
Variable	Coefficient	Std. Error	t-Statistic	Prob.
C	62.49124	10.68910	5.846259	0.0043
SSQD	−3.053383	1.058903	−2.883535	0.0449
SCMJ	0.091875	0.017341	−5.298166	0.0061
AR(2)	−0.368923	0.520994	−0.708113	0.0180
R-squared	0.884371	Mean dependent var		3.105800
F-statistic	10.19776	S.D. dependent var		3.109918
Prob(F-statistic)	0.024081	Durbin-Watson stat		2.010931

**Table 10:** Estimation of the fishing vessel damage equation measurement model.

Dependent Variable: SHYC				
Variable	Coefficient	Std. Error	t-Statistic	Prob.
C	12788.52	220977.1	0.057873	0.0591
SSQD	−2479.115	672.0659	−3.688797	0.0663
YCZS	0.097319	4.050583	−0.024026	0.0030
MA(3)	−0.986522	0.047513	−20.76325	0.0023
R-squared	0.985664	Mean dependent var		1577.667
F-statistic	45.83714	S.D. dependent var		1838.775
Prob(F-statistic)	0.021426	Durbin-Watson stat		1.967204

**Table 11:** Estimated results of the econometric model of the affected population.

Dependent Variable: SZBZ				
Variable	Coefficient	Std. Error	t-Statistic	Prob.
C	0.296953	0.040967	7.248607	0.0008
SSQD	−0.028849	0.013821	−2.087308	0.0012
RKMD	0.000322	2.61 × 10^−5^	−12.36038	0.0001
AR(2)	−0.815130	0.272247	−2.994080	0.0303
MA(1)	−0.999229	0.274914	−3.634697	0.0150
R-squared	0.933918	Mean dependent var		0.038245
F-statistic	17.66600	S.D. dependent var		0.032020
Prob(F-statistic)	0.003743	Durbin-Watson stat		2.002997

**Table 12:** Estimation results of the breakwater damage equation measurement model.

Dependent Variable: FBDH				
Variable	Coefficient	Std. Error	t-Statistic	Prob.
C	180.2115	937.7359	0.192177	0.0099
SSQD	−48.72474	4.731625	10.29768	0.0020
AR(1)	0.911915	0.327740	2.782434	0.0089
MA(2)	−0.999561	0.090380	−11.05949	0.0016
R-squared	0.961123	Mean dependent var		38.16714
F-statistic	24.72222	S.D. dependent var		53.60334
Prob(F-statistic)	0.012860	Durbin-Watson stat		1.892249

**Table 13:** Damage to the housing equation measurement model estimation results.

Dependent Variable: SHFW				
Variable	Coefficient	Std. Error	t-Statistic	Prob.
C	5.362865	1.234981	4.432468	0.0225
SSQD	−1.475384	0.411115	−3.588740	0.0371
MA(3)	−0.935312	0.072827	−12.84300	0.0010
R-squared	0.887513	Mean dependent var		0.582300
F-statistic	11.83491	S.D. dependent var		1.148941
Prob(F-statistic)	0.037727	Durbin-Watson stat		1.229312

**Table 14:** Estimation of the results of the direct economic loss equation.

Dependent Variable: ZSBZ				
Variable	Coefficient	Std. Error	t-Statistic	Prob.
C	0.005963	0.001035	5.763238	0.0012
QD	−0.001699	0.000383	−4.442445	0.0044
JJMD	0.001908	0.000845	−2.257594	0.0648
AR(1)	−0.952453	0.121304	−7.851760	0.0002
MA(2)	−0.956248	0.052066	−18.36623	0.0000
R-squared	0.875984	Mean dependent var		0.000807
F-statistic	10.59518	S.D. dependent var		0.000921
Prob(F-statistic)	0.006920	Durbin-Watson stat		1.554477

**Table 15:** Prediction of storm surge disasters based on support vector machines (unit: $100 million).

Forecast Sample	Sample 1	Sample 2	Sample 3
Direct economic (original loss value)	23.70	18.89	15.22
Direct economic (predictive loss value)	21.12	18.34	15.41
